# Radiation Therapy-Induced Tumor Invasiveness Is Associated with SDF-1-Regulated Macrophage Mobilization and Vasculogenesis

**DOI:** 10.1371/journal.pone.0069182

**Published:** 2013-08-05

**Authors:** Shu-Chi Wang, Ching-Fang Yu, Ji-Hong Hong, Chien-Sheng Tsai, Chi-Shiun Chiang

**Affiliations:** 1 Department of Biomedical Engineering and Environmental Sciences, National Tsing-Hua University, Hsinchu, Taiwan; 2 Department of Radiation Oncology, University of Texas Southwestern Medical Center, Dallas, Texas, United States of America; 3 Department of Radiation Oncology, Chang-Gung Memorial Hospital, Taoyuan, Taiwan; 4 Department of Medical Imaging and Radiological Science, Chang Gung University, Taoyuan, Taiwan; University of Florida, United States of America

## Abstract

Radiation therapy (RT) remains the front-line treatment for high-grade gliomas; however, tumor recurrence remains the main obstacle for the clinical success of RT. Using a murine astrocytoma tumor cell line, ALTS1C1, the present study demonstrates that whole brain irradiation prolonged the survival of tumor-bearing mice, although the mice eventually died associated with increased tumor infiltration. Immunohistochemical (IHC) analysis indicated that RT decreased the microvascular density (MVD) of the primary tumor core, but increased the MVD of the tumor invasion front. RT also increased the number of tumor-associated macrophages (TAMs) and the expression of stromal cell-derived factor-1 (SDF-1) and hypoxia-inducible factor-1 (HIF-1) at the tumor invasion front. SDF-1 expression suppressed by siRNA (SDFkd tumors) showed a decrease in RT-enhanced tumor invasiveness, leading to prolonged survival of mice bearing these tumors. The invasion front in SDFkd tumors showed a lower MVD and TAM density than that in the islands of the control or irradiated ALTS1C1 tumors. Our results indicate that tumor-secreted SDF-1 is one key factor in RT-induced tumor invasiveness, and that it exerts its effect likely through macrophage mobilization and tumor revascularization.

## Introduction

Glioblastoma multiforme (GBM) is the most frequent type of malignant brain tumor in adults and continues to be a serious clinical problem [1]. Unlike the huge improvement in the prognosis of other tumors, such as breast cancer, melanoma, and prostate cancer, the improvement in the cure rate of brain tumors in the past 10 years has remained low [2]. Although the patient's survival rate depends on the histological grade of the tumor, glioma cell invasion into adjacent normal brain regions is believed to be the major reason for the failure of these tumors to respond to treatments such as neurosurgery, radiation therapy (RT), and chemotherapy.

RT has been an essential part of treatment for most glioma patients and can extend the median survival by 6–8 months; however, it eventually fails due to recurrence of the tumor [3]. Despite the intrinsic radioresistance exhibited by most glioma cells, glioma invasiveness is another challenge during RT, because radiation, in contrast to the expected cytotoxic effects, might also enhance glioma cell migration and invasion [4], [5]. It has been reported that most relapse occurs in the target volume of RT [6], [7]. Expression of several candidate genes, such α_v_β3 integrin and the matrix metalloproteinases, MMP-2, and MMP-9, in tumor cells has been reported to be associated with radiation-induced glioma cell invasion [5], [8].

Invasion is a process of the cross-talk between the invaders and the host. The advances in genomics and proteomics have provided many glioma invasion-associated candidate genes [9]–[11]. Factors that possess antocrine activity can promote glioma invasion and at the same time they act as paracrine mediators to remodel the brain microenvironment to favor invasion. These factors are particularly important targets for the study of glioma invasion [12]. Among these factors, stromal cell-derived factor-1 (SDF-1/CXCL12) is known to be involved in glioma invasion by recruiting macrophage or T-regulatory cell migration to the peritumoral area [13], by cross-talking with endothelial cells [14], or by mobilizing hematopoietic stem cells and progenitor cells [15]. Although low levels of SDF-1 are usually detected in neutrons, astrocytes, and microglia, SDF-1 expression in the brain tumor increases with increasing tumor grades [16]. This may be associated with the hypoxia-induced expression of SDF-1 and its ability to enhance the survival of cells under hypoxic conditions [17], [18].

Studies have shown that the blockage of the interaction of SDF-1 with its receptor, CXCR4, by a clinical drug, AMD3100, enhanced the efficacy of RT in glioma [19], breast, and lung carcinoma [20] by preventing the recruitment of bone marrow-derived cells (BMDCs) and tumor revascularization. Our previous study in a murine astrocytoma tumor cell line, ALTS1C1 [18] demonstrated that SDF-1 expression was higher in the invading tumor front than that in the primary tumor core, and that the level of SDF-1 expression was associated with tumor invasiveness. In this study, we further explored the role of tumor-secreted SDF-1 in radiation-induced macrophage migration, tumor vascularization, and tumor invasiveness.

## Materials and Methods

### Ethics statement

C57BL/6J mice were purchased from the National Scientific Council, Taiwan. This study was carried out in strict accordance with the recommendations in the Guide for the Care and Use of Laboratory Animals of the National Tsing Hua University, Taiwan. The protocol was approved by the Institutional Animal Care and Use Committee (IACUC approval number: 09929) of National Tsing Hua University, Taiwan. All surgery was performed under sodium pentobarbital anesthesia, and all efforts were made to minimize suffering.

### Cell lines, irradiation, and colony assay

ALTS1C1 was derived from primary astrocytes transformed by SV40 large T antigen and serial *in vivo* passage [18] and is deposited in Bioresource Collection and Research Center (BCRC-60582), Taiwan. Cells were irradiated in log phase using a cobalt source with a dose rate of 50 cGy/minute in the Nuclear Science and Technology Development Center, National Tsing Hua University, Taiwan. After exposure, varying cell numbers were seeded in triplicate into 100 mm culture dishes within 10 ml regular cell culture medium and incubated for 7–14 days. Colonies were counted after staining with 3% Giemsa solution (Merck, KGaA, Germany) and the survival fraction determined.

### Tumor implantation and tissue processing

To establish intracranial (i.c.) tumors, ALT-S1C1 glioma cells were implanted into the brains of 8- to 10-week-old C57BL/6 mice as described in previous publication [18]. The mice were killed by day 21 or when the neurologic syndrome was obvious. After mice were killed by CO_2_ inhalation, cardiac perfusion was performed with 4% paraformaldehyde in 1X PBS to fix the tissues. The tissues were either embedded in paraffin for regular microtome section or OCT medium for frozen section. The tissues were sliced into 4 µm or 10 µm for paffaffin or frozen section, respectively. Tumors were measured grossly in three orthogonal axes to determine tumor volumes. The hematoxylin and eosin (H&E)-stained maximal coronal sections were used as a measurement of tumor cross section area ((the radius of long axis + the radius of short axis)/2)^2^ x π) and the examination of overall tumor histopathology.

### Tumor irradiation

Mice were anesthetized by pentobarbital anesthesia and restrained by adhesive tape during irradiation. Irradiation field (1 cm) was limited to the area between the ears and eyes. The head was covered with 1 cm bolus and irradiated by 6-MV X-rays from a linear accelerator with a dose rate of 2 to 3 Gy/min. Single dose was given on day 13 after brain tumor implantation.

### Immunohistochemistry

Tissues embedded in OCT were used for IHC staining. Frozen tumors were sectioned (10 μm), mounted onto slides and maintained at −20^o^C. To perform IHC staining, tissues were fixed with methanol and permeabilized in 0.01% Tween-20/0.1% Triton X-100 in PBS. Slides were blocked with blocking buffer (4% fetal bovine serum-FBS and 1% normal serum of secondary antibody host) for 30 minutes at room temperature. The following antibodies were used: rabbit anti-mouse Iba-I (polyclonal; Wako, Osaka, Japan), rat anti-mouse CD31 (Clone MEC13.3; BD Pharmingen), rat anti-mouse F4/80 (Clone CI:A3-1; AbD Serotec, Oxford, England), and rat anti-mouse CD68 (Clone FA-11; BD Pharmingen). Slides were washed and incubated with anti-corresponding host of secondary antibodies conjugated with Alexa Fluor 488 or Alexa Fluor 594 (Invitrogen) for 2 hours. The sections were washed and mounted with DAPI (5 μg/mL; Invitrogen) to visualize nuclei.

Microvascular density (MVD) was determined as the number of pixels positive for CD31 divided by the selected tumor island area. The density of macrophages in the tumor island was defined as the pixels positive for CD68 divided by total DAPI number in the selected tumor area. Images were viewed and captured by an AxioCam MRC-5 camera on an Axiovert40 fluorescence microscope (Carl Zeiss, Inc., Göttingen, Germany) or Laser Scanning Confocal microscope (LSM5, Zeiss, Germany) and processed using Image-pro plus 6.0.

The invasive pattern was assessed on whole brain serial sections by DAPI stain. The thickness of each section was 10 µm and every 10^th^ section was used to count the tumor invading islands. An island was scored as positive when the value of ((island length + island width)/2) ≤200 µm and each island was not connected to the main tumor edge.

### Immunoblotting

Western blot analysis was performed with 20 μg protein extracts separated by 10% polyacrylamide gel and probed by rabbit anti-mouse SDF-1α polyclonal antibody (1∶1000; eBioscience, San Diego, CA, USA), anti-mouse HIF-1α monoclonal antibody (1∶500; Clone H1alpha67; Novus, Littleton, CO, USA) or an anti-actin antibody (1∶5000; Clone ACTN05; Thermo, Fremont, CA, USA) as loading control.

### ELISA

The secreted SDF-1 levels in the culture supernatants were determined in triplicate using an SDF-1 ELISA kit (R&D Systems, Minneapolis, MN, USA) and normalized to total cell numbers at the end of the treatment. ELISA was performed according to the instruction provided by the manufacturer.

### Statistics

Statistical analyses were performed using the 2-tailed Student's *t* test or one-way ANOVA to determine statistical significance. *P* values (exact significance) of less than 0.05 were considered statistically significant. All calculations were performed using GraphPad Prism 5.

## Results

### Local brain irradiation shrinkages tumor volume but increases the number of infiltrating islands

**Figure 1:**
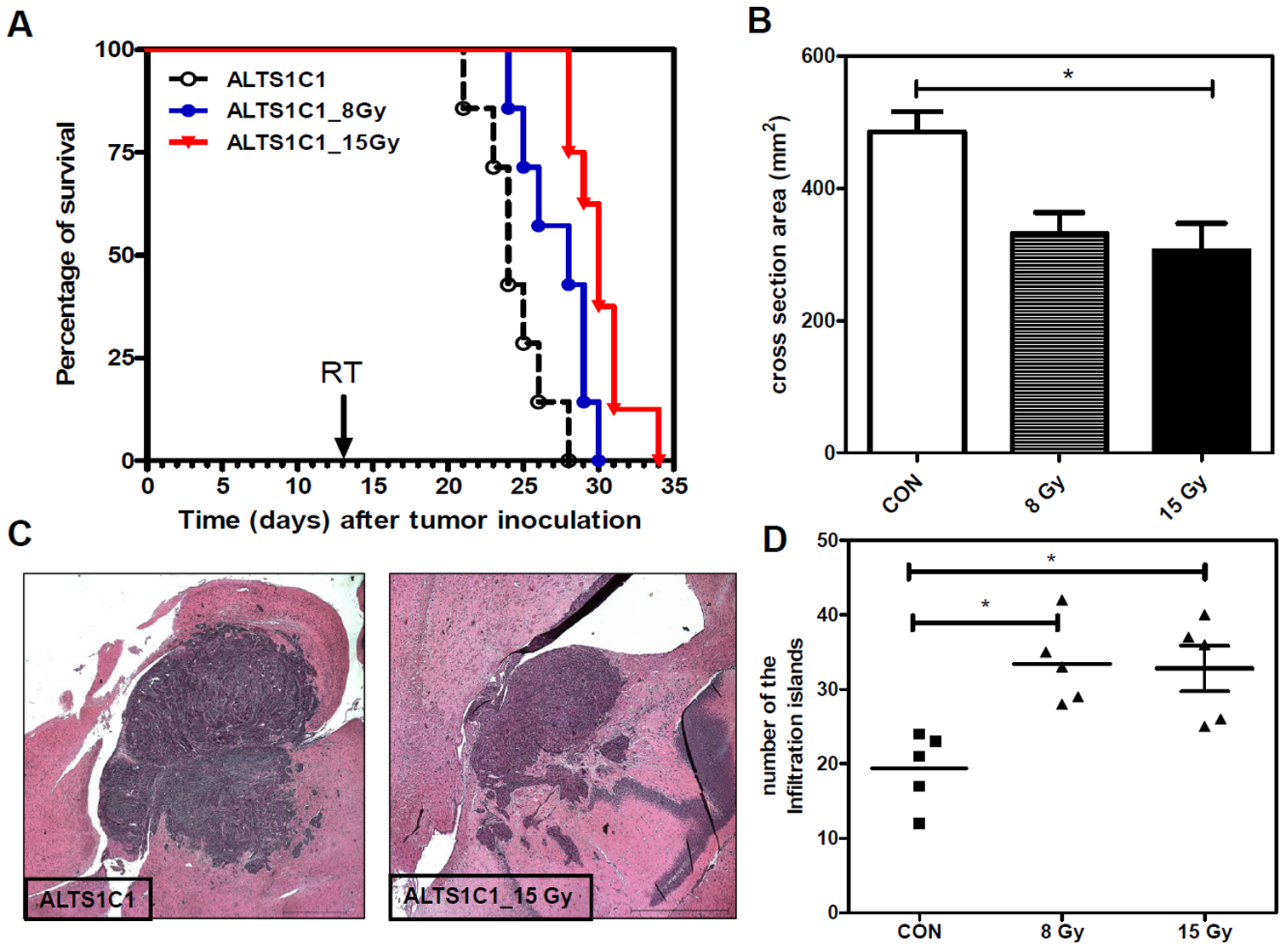
Local brain irradiation prolonged the survival of tumor-bearing mice, but mice eventually died due to increased tumor. (A) Kaplan-Meier survival curves of ALTS1C1 glioma-bearing mice after 8 Gy or 15 Gy single-dose irradiation. The arrow indicates the time (day 13 after brain tumor implantation) at which radiation was given. (B) The mean diameter of control and irradiated ALTS1C1 brain tumors measured at the maximum cross-section in H&E-stained tissues. (C) Low-magnification merged image showing tumor invasion into the adjacent brain tissues. Scale bar  = 1 mm. (D) Scatter plot of the invasion islands in the ALTS1C1 control and single-dose irradiated brain tumors. The invasive pattern was assessed in whole-brain serial sections by DAPI staining. The plot represents the average island number of each brain in each group, as calculated using a microscope (×100 power). * p<0.05.

To examine the *in vivo* response of ALTS1C1 tumor cells to radiation, mice transplanted with ALTS1C1 tumors received local brain irradiation with a single dose of 8 Gy or 15 Gy at day 13 after intracranial implantation. Kaplan-Meier analysis showed that the medium survival time of tumor-bearing control mice was 24±2 days (Figure 1A). A single dose of 8 Gy and 15 Gy irradiation prolonged the medium survival day to 28±2 and 30±1 days, respectively. An analysis of the tumor volume was obtained by measuring the maximum cross-sectional area base on H&E stained-sections from sick mice showed that both 8 Gy and 15 Gy significantly reduced the primary tumor size to approximately 68% and 64%, respectively, of the control tumors (Figure 1B). However, the histological analysis of the sections revealed numerous infiltrating islands in the irradiated tissues (Figure 1C and Figure S1). Quantification of the number of islands from the largest cross-section of each tumor showed that the number of islands was significantly increased in the irradiated group (Figure 1D). These results indicated that local brain irradiation effectively reduced the growth rate of the primary tumor, but promoted tumor invasiveness, which might increase the complexity of glioma following radiation therapy.

### A single dose of whole-brain irradiation increases microvascular and microglia/macrophage density at the invading tumor front

**Figure 2:**
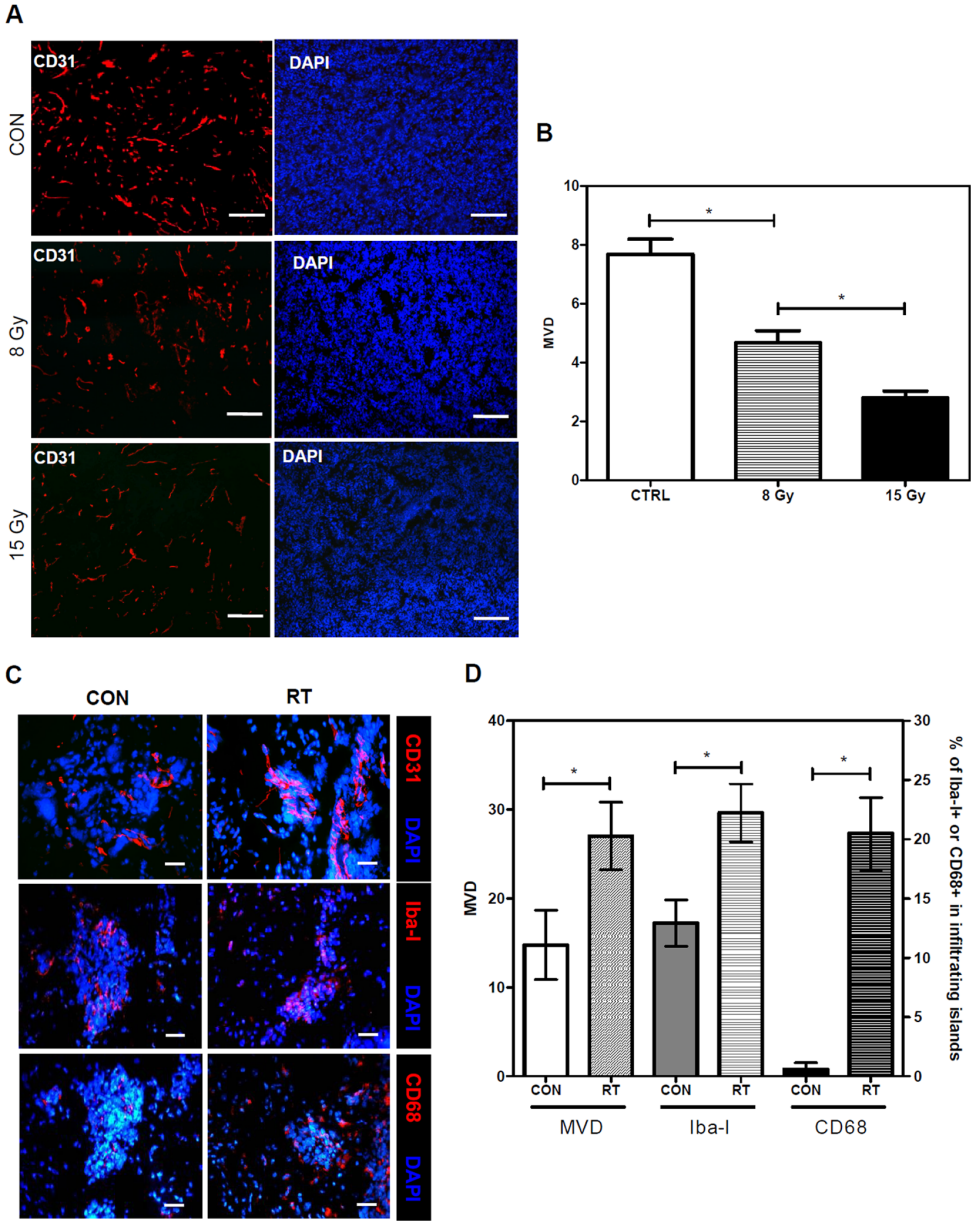
Irradiation increased the MVD and TAM density in the infiltrating islands. (A) IHC staining of the vessels by CD31 antibody (red) and of the nuclei by DAPI (blue) in the tumor core region of the control, 8 Gy, or 15 Gy single-dose irradiated ALTS1C1 tumors. Scale bar  = 100 μm. (B) Quantification of MVD in CD31-positive cells in the brain tumor core region (n = 6∼12 fields per tumor, with a total of five tumors in each group; * p<0.05). (C) Confocal imaging of IHC-stained sections for detection of CD31 (red), Iba-1 (red), CD68 (red), and nuclei staining by DAPI (blue) in infiltrating islands of control and irradiated ALTS1C1 tumors. Scale bar  = 20 μm. (D) Quantification of MVD and cells expressing Iba-1, and CD68 in the infiltrating islands of control or single-dose irradiated ALTS1C1 tumors (n = 20 islands per tumor, with a total of three tumors in each group; * p<0.05).

A previously study of ALTS1C1 tumor [18] demonstrated that the invading tumor front exhibited a higher microvascular density (MVD) and expressed higher levels of F4/80-positive microglia/macrophages than in the primary tumor core. Our earlier study in a TRAMP-C1 prostate tumor model also showed that RT can decrease MVD and alter the spatial distribution of tumor-associated macrophages (TAMs) [21]. The present study further confirmed that RT significantly decrease MVD in the main tumor core (Figures 2A and 2B), but unexpectedly increase MVD in the invasion front of ALTS1C1 brain tumors (Figure 2C and 2D). The invading tumor front of a tumor usually contains two parts, the edge of the primary tumor core and the infiltrating islands. The former can be easily identified by 4′, 6-diamidino-2-phenylindole (DAPI) nuclear staining; however, its range cannot be clearly defined. The infiltrating island can be objectively defined, as described in the Methods section. For the purpose of comparison, all quantitative data only recorded the events in infiltrating islands.

The density of CD68-positive macrophages in the hypoxic region of the primary tumor core was also increased after RT [22]. Immunohistochemical (IHC) analysis of the resident microglia and infiltrating macrophages in the infiltrating island using the macrophage differentiation markers Iba-Ι and CD68 showed that both these cell types were also significantly increased after RT (Figures 2C and 2D). However, more dramatic changes were found in the CD68-positive macrophages; there were fewer CD68-positive macrophages than Iba-I-positive macrophages in the islands of control tumors; however, CD68-positive macrophages reached a number similar to Iba-I-positive macrophages after RT. These results indicated that infiltrating islands in irradiated ALTS1C1 brain tumors have un-expectedly high MVD and infiltrating macrophages.

### The origin of macrophages in ALTS1C1 brain tumors

**Figure 3:**
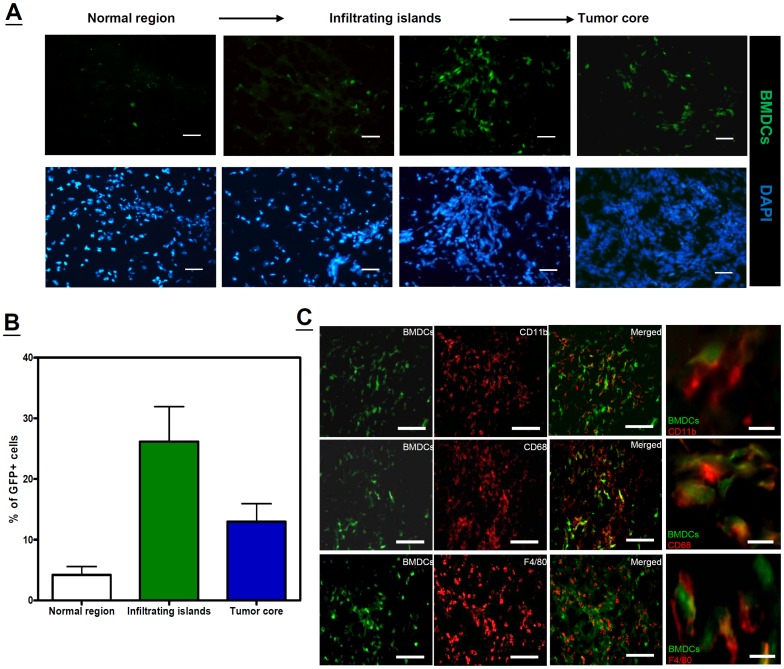
Peripheral myeloid cells infiltrate into the invading brain tumor. (A) Migration of GFP-BMDCs in the ALTS1C1 brain tumor sections from adjacent normal regions to the tumor core. Cell nuclei were stained with DAPI. All scale bars: 50 μm. (B) Percentage of GFP-BMDCs in normal brain region, islands, and different areas of the tumor core in ALTS1C1 brain tumor (n = 10–15 fields per area in total 3 tumors). (C) IHC counterstaining of GFP-positive cells (green color) with CD11b (red color of top panel), CD68 (red color of middle panel), and F4/80 (red color of bottom panel) antibodies in ALTS1C1 brain tumors obtained from mice receiving GFP-BM transplantation. Low power scale bar (left 3 columns): 100 μm. High power scale bar (right column): 20 μm.

To examine the hypothesis that an RT-induced increase in TAM density in the infiltrating islands was the result of a macrophage infiltration from the peripheral blood. ALTS1C1 tumors grown in green fluorescent protein-tagged bone marrow (GFP-BM)-transplanted mice were used to investigate this (Figure S2A). Only a small number of GFP-positive cells were found in the control or phosphate-buffered saline (PBS)-injected brain sections **(** Figure S2B **)**. In contrast, numerous GFP-positive cells were recruited into the ALTS1C1 tumor region, especially in the tumor fronts and invasive islands (Figure 3A). The percentage of GFP-positive cells in the normal brain, invasive islands, and tumor core region was 4.0%±1.4%, 27.1%±5.7%, and 13.1%±2.9%, respectively (Figure 3B). When the tumor samples were further analyzed by flow cytometry using triple color staining for CD11b, CD45, and GFP, results showed that 73.0%±6.3% of CD45^hi^/CD11b^hi^ macrophages co-expressed GFP, but only 10.1%±2.9% of CD45^mid^/CD11b^hi^ microglia co-expressed GFP (Figures S2C and S2D). IHC analysis using double staining further confirmed the co-expression of GFP in CD11b-positive, F4/80-positive, and CD68-positive macrophages (Figure 3C). These results indicated that the ALTS1C1 brain tumors contain a large amount of infiltrating macrophages that migrate from circulating monocytes.

### The contribution of tumor-secreted SDF-1 in RT-induced tumor invasiveness

**Figure 4:**
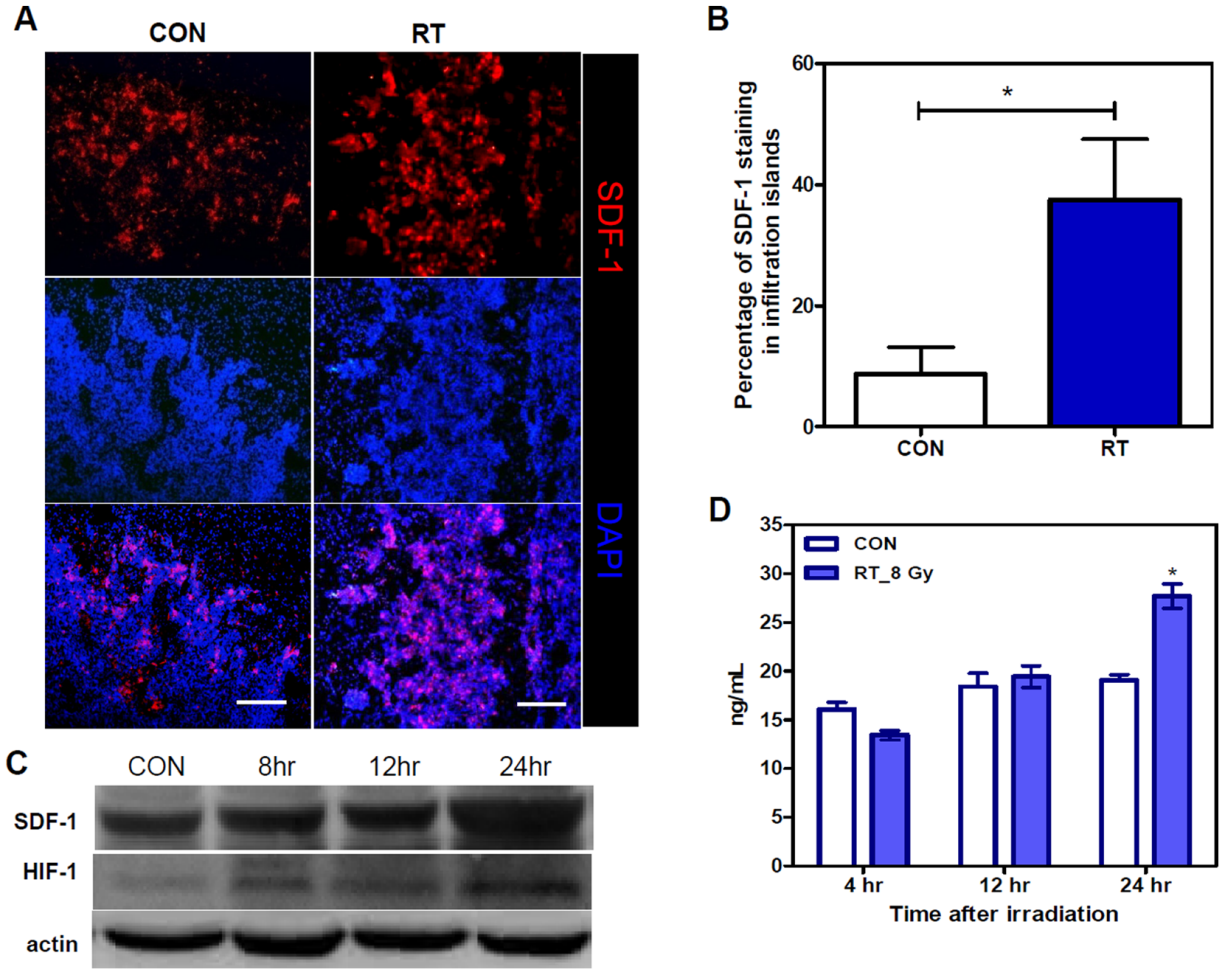
Irradiation increased SDF1 expression in infiltrating tumor islands. (A) IHC staining of SDF-1 (red) and nuclei by DAPI (blue) in the control (CON) and in 8 Gy (RT) irradiated brain tumors. Scale bar  = 200 μm. (B) Quantification of SDF-1 expression in selected tumor island areas. * p<0.05. (C) Western blot analysis to analyze the expression of SDF-1 and HIF-1 proteins in ALTS1C1 control or single-dose 8 Gy-irradiated cells. (D) ELISA results of SDF-1 production by ALTS1C1 control or single-dose of 8 Gy-irradiated cells. * p<0.05.

Radiation-induced increases in the number of macrophage both in the tumor core and at the invading tumor front is consistent with the general view on this subject; however, increased MVD in the irradiated infiltrating islands, in contrast to the decreased MVD in the irradiated tumor core, was an un-expected result. The cause of this unusual finding is unclear. Many studies have shown that SDF-1 is not only a chemoattractant for macrophages [18], [23], [24], but that it also contributes to tumor vessel formation [25]–[27]. In the present study, IHC staining showed that the expression of SDF-1 was higher in the invading tumor front than that in the primary tumor core [18] and that SDF-1 expression in the invading tumor front was further increased after RT (*p = 0.0105*) (Figures 4A and 4B). Because the invading tumor front showed a high MVD, we questioned if RT could induce SDF-1 expression. Western blot analysis (Fig. 4C) and enzyme-linked immunosorbent assay (ELISA; Figure 4D) investigations of ALTS1C1 cells irradiated *in vitro* by 8 Gy showed a substantial increase in SDF-1 expression at 24 h after irradiation. This result indicated that RT-induced SDF-1 expression may be responsible for the increase in MVD and the number of infiltrating macrophages after irradiation. An increase in HIF-1 expression, as indicated by Western blot analysis (Figure 4C) also showed that an increase in HIF-1 expression is induced as early as 8 h after irradiation. The expression of HIF-1 in the invading tumor front of both the control and irradiated tumors was confirmed by IHC staining (Figure S3).

**Figure 5:**
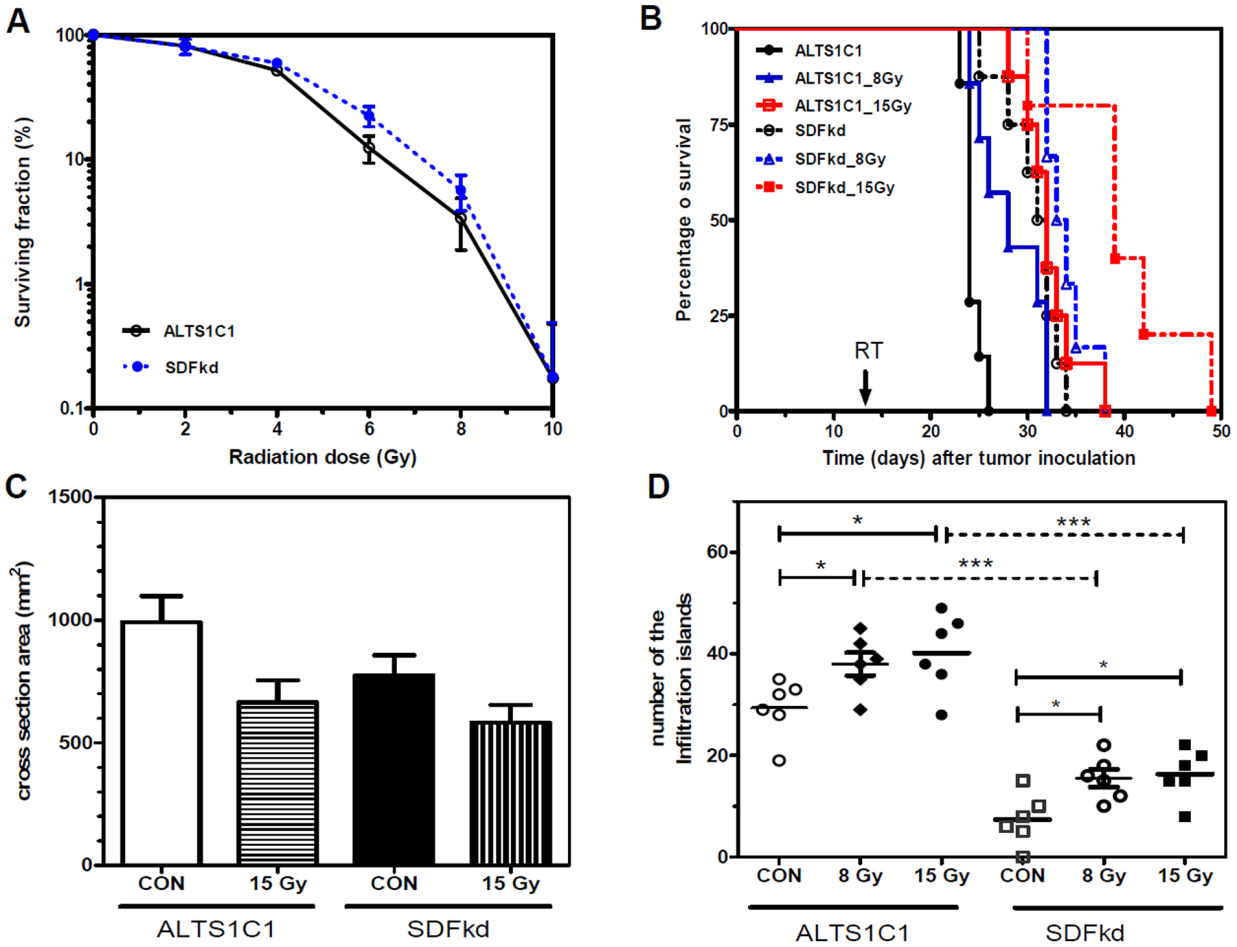
*In vitro* and *in vivo* radiation response of ALTS1C1 and SDFkd tumors. (A) The survival curve following IR was assayed by clonogenic assay. (B) Kaplan–Meier survival curves of ALTS1C1 and SDFkd tumor-bearing mice treated with various doses of irradiation. The arrow indicates the time (day 13 after brain tumor implantation) for which radiation was given. (C) The mean diameter of control and single-dose irradiated ALTS1C1 or SDFkd brain tumors measured at the maximum cross-section in H&E-stained tissues. * p<0.05. (D) Scatter plot of tumor islands in control and single-dose irradiated ALTS1C1 and SDFkd brain tumors. The invasive pattern was assessed in whole brain serial sections by DAPI staining. The plot represents the average island number of each brain in each group, as calculated using a microscope (× 100 power). * p<0.05, *** p<0.001.

A siRNA approach that was used to suppress SDF-1 expression in ALTS1C1 cells demonstrated that tumor-secreted SDF-1 contributed to glioma invasiveness [18]. This study further explored if RT-induced SDF-1 expression in ALTS1C1 cells plays a role in RT-induced tumor invasiveness. In this present study, we first examined if the suppression of SDF-1 expression by siRNA affected the cell response to irradiation. An i*n vitro* clonogenic assay demonstrated that the ALTS1C1 and SDFkd cells have the same response to irradiation (Figure 5A). Our *in vivo* study not only re-confirmed that SDFkd tumor-bearing mice survived longer than ALTS1C1 tumor-bearing mice [18], but also demonstrated that the combination of SDF-1 inhibition and RT could further prolong survival of the mice (Figure 5B). Using the maximum cross-sectional area as an index for tumor size, we further confirmed that irradiation could effectively shrink the size of the primary tumor, and this effect was independent of the SDF-1 levels (Figure 5C). Furthermore, the suppression of SDF-1 expression not only reduced the number of infiltrating islands in control tumors [18], but also in irradiated tumors (Figure 5D, p<0.001). However, it appeared that radiation could also promote the development of infiltrating islands in SDFkd tumors. Two possibilities are suggested in this regard: (1) This may be caused by the minimal SDF-1 secreted by SDFkd tumors or TAMs [18]; (2) SDF-1 is one of factors that are involved in tumor invasiveness. Nevertheless, our results indicated the important role of SDF-1 on tumor invasiveness of both control and RT-associated tumor recurrence. This data also indicates that tumor-bearing mice died is a complex result of primary tumor size and the number of infiltrating islands. When the tumor invasion is suppressed, such as the case in SDFkd mice, tumor size is the main factor responsible for mice death.

### Tumor-secreted SDF-1 expression is associated with an RT-dependent increase in MVD and TAM density in invading tumors

**Figure 6:**
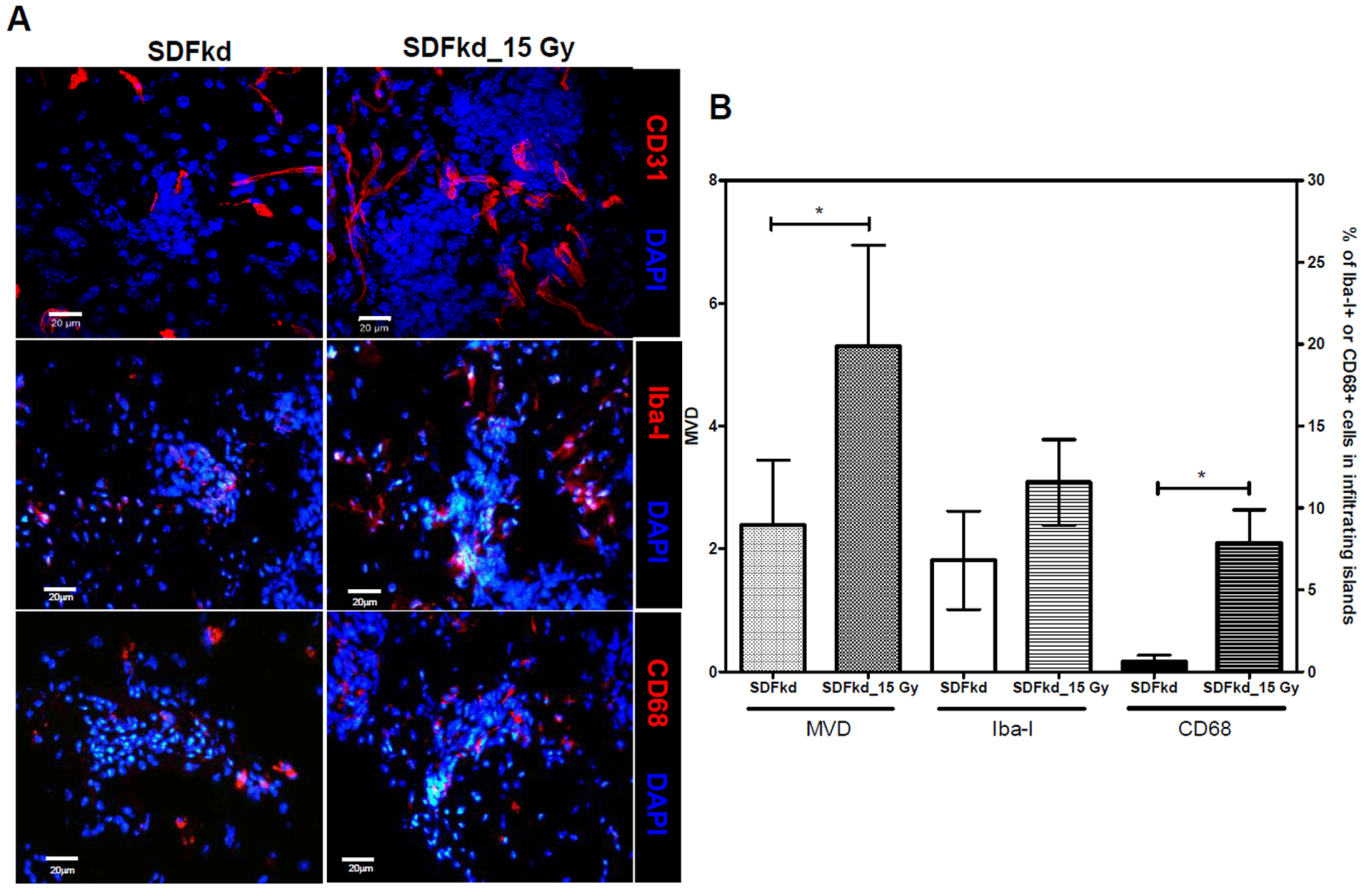
Tumor MVD and TAM density in SDFkd tumor following irradiation. (A) Confocal imaging of IHC staining for CD31 (red), Iba-1 (red), CD68 (red), and nuclei by DAPI (blue) on the infiltrating islands of SDFkd control or 15 Gy-single dose irradiated SDFkd tumors. Scale bar  = 20 μm. (B) Quantification of the change of MVD, Iba-1, and CD68 in brain tumor islands (n = 20 islands per tumor, with a total of three tumors in each group; * p<0.05).

The IHC results showed that the MVD and TAM density in the invading tumor front of the SDFkd tumors significantly decreased to 16% and 55%, respectively (Fig. 6), of the control tumors (Figure 2D). Irradiation also increased MVD and CD68-positive macrophages, but did not increase the density of Iba-I-positive microglia in SDFkd tumors (Figure 6B). The numbers of CD68-positive macrophages and Iba-I-positive microglia in the SDFkd tumors were lower (20% and 38%, respectively) than those in the irradiated ALTS1C1 tumor (Figure 2D). These data indicated that down regulation in the expression of tumor-secreted SDF-1 leads to a decrease in MVD and TAM density in the invading tumor. The increase of MVD and TAM density in irradiated SDFkd tumors is likely the result of minimal SDF-1 secretion by SDFkd tumors or other stroma cells.

## Discussion

The present study demonstrated that irradiation has the ability to promote tumor invasive behavior, particularly at the invading tumor front. The radiation-induced increase in tumor invasiveness and number of infiltration islands play a significant role on glioma failure following RT. It has been previously demonstrated in a ALTS1C1 tumor model [18] that the invading tumor front has a distinct microenvironment from that of the primary tumor core, such as a different ratio of the TAM subtypes and a higher MVD associated with higher levels of vascular endothelial growth factor receptor-1 (VEGFR-1) and SDF-1 expression. Our study specifically focused on the response of the invading tumor front to RT. This study added that whole-brain irradiation further increased MVD and the number of infiltrating macrophages at the invading tumor front in ALTS1C1 tumor cells. It is possible that these islands were the left behind shrinking tumors. If this is the case, the vessel density following irradiation is expected to be lower as seen in primary tumor core (Fig. 2A and 2B). However, the vessel density of these islands was further increased after RT (Fig. 2C and 2D), indicating they were invasive islands. We further demonstrated that ALTS1C1 tumors in which SDF-1 expression was suppressed by siRNA (SDFkd tumors) showed a decrease in RT-enhanced tumor invasiveness, leading to prolonged survival of mice bearing these tumors.

Our finding that RT can enhance glioma invasiveness cautions the use of current clinical protocol of involved-field radiation therapy for glioma. We demonstrated increased MVD in the irradiated infiltrating islands of the tumor-invading front, which was an unexpected result. Most studies to date have focused on the response of the primary tumor core to radiation. A recent report by Wild-Bode, C. et al [5] demonstrated that radiation-induced glioma invasiveness was associated with the synergistic interaction of the altered BCL-1/BAX rheostat, which favored the resistance of glioma cells to apoptosis and the increased expression of migration/invasion-associated genes, such as α_v_β3 integrin, MMP-2, and MMP-9. Our study showed that the primary tumor core and invading tumor front displayed different response behaviors to RT, probably due to the fact that both have different tumor microenvironments. Although RT increased the TAM density in both the primary tumor core and the tumor-invading front, the response of TAMs to RT-induced microenvironmental changes in the primary tumor core and the tumor-invading front is different. We have previously demonstrated that an RT-induced increase in TAMs in the primary tumor core, whether it is a prostate tumor or a glioma, is mainly associated with the development of RT-induced chronic hypoxia [22]. However, RT-induced increase in TAMs at the tumor-invading front may be caused by a mechanism other than chronic hypoxia, because this region showed higher MVD. This may be associated with the different composition of TAMs in the primary tumor core and the invading tumor front. We have previously shown that a majority of the TAMs in the primary tumor core are double positive for the CD68 and F4/80 markers, and the TAMs in the invading tumor front are both CD68 and F4/80 double-positive as well as F4/80-positive and CD68-negative TAMs [18]. This result is consistent with the higher Iba-1 expression observed at the invading tumor front compared with CD68 expression, indicating that a greater number of mature macrophages or activated resident microglia are involved in the invading tumor front. Our results suggest that RT recruits the infiltration of more CD68-positive macrophages from peripheral blood. These RT-recruited CD68-positive macrophages may act as M2 macrophages to promote tumor invasion. RT-mediated increase in MVD at the invading tumor front was another unique feature demonstrated by our study, given that the MVD of the primary tumor core decreased after RT. In fact, most studies have demonstrated a reduction in MVD after RT [21], [28]. Taken together, our data suggest that certain factors associated with the invading tumor front govern radiation-induced invasiveness of the glioma.

Among factors that are associated with glioma invasiveness [12], SDF-1 seems particularly important in ALTS1C1 tumors, because the suppression of SDF-1 expression in ALTS1C1 tumors by siRNA was able to decrease MVD, TAM density, and tumor invasiveness. In the present study, we found higher SDF-1 expression in the invading tumor front before irradiation, and it was further up regulated after irradiation. A previous study showed that 15 Gy of whole brain radiation to U251 GBM bearing-nude mice transiently increased SDF-1 expression up to 3 months in the irradiated primary tumor [19]. Increased SDF-1 expression in these irradiated primary tumors was mainly the result of radiation-induced hypoxia-associated HIF-1 expression. Although irradiation by itself can induce HIF-1 expression in tumors [29], HIF-1 expression in irradiated primary tumors was primarily a result of tumor hypoxia [21], [30], [31]. Radiation-induced SDF-1 expression in the invading tumor front of ALTS1C1 tumors was also correlated with the increase in HIF-1 [32]; however, interestingly, this region had a higher MVD than the primary tumor core. This is perhaps another piece of direct *in vivo* evidence that demonstrates that radiation can induce HIF-1 expression [29] and leads to SDF-1 expression [19]. Although we do not have HIF-1 knockout mice to prove this pathway, our *in vitro* Western blot results show that radiation-induced HIF-1 expression in ALTS1C1 cells was observed earlier than SDF-1 expression, which particularly supports the above view.

It appears that radiation-induced SDF-1 expression is one of factors responsible for the radiation-induced increase in MVD and infiltrating macrophages, which may be subsequently responsible for radiation-induced tumor invasiveness. This is because the suppression of SDF-1 expression by siRNA did cause a decrease in these radiation-induced phenomena occurring at the invading tumor front. Kioi et al. have recently shown that the administration of AMD3100, an inhibitor of the SDF-1/CXCR4 interaction, blocked the recurrence of U251 GBM in nude mice after RT [19] by preventing the recruitment of radiation-induced BMDCs and by inhibiting vasculogenesis. This study supports our finding in irradiated SDFkd tumors, which showed a decrease in MVD. Although, treatment with AMD3100 appears to be a better strategy than SDF inhibition by siRNA to enhance the efficacy of RT, given that the former strategy can cure tumors, it may not be appropriate to compare the AMD3100 study to ours, because different cell lines (U251 human GBM versus ALTS1C1 murine astrocytoma) and different mice models (nude mice versus C57BL/6J mice) were used. Nevertheless, these two studies demonstrate that radiation-induced SDF-1 expression is one of important factors in radiation-induced tumor invasiveness, and it exerts its effect through macrophage mobilization and vessel vascularization. It needs to remind that other pathways, such as CSF1/CSF1R, may also be involved in macrophage mobilization as demonstrated in other tumor models [33]–[35].

In conclusion, this study demonstrated that tumor-secreted SDF-1 is one of factors responsible for RT-increased tumor invasiveness and acts by regulating macrophage recruitment and vessel vascularization. Thus, our study may add a clinically relevant element to the interpretation of the efficacy of radiotherapy.

## Supporting Information

Figure S1 **Histopathology of control (un-disturbed tumor) versus irradiated tumor (A) DAPI stain on frozen section and (B) H&E stain on paraffin section on control or 8 Gy single dose-irradiated ALTS1C1 brain tumors.** Scale bar  = 100 μm.
(DOC)

Figure S2 **The origin of macrophages in ALTS1C1 brain tumors.** (A) Schema of the GFP- bone marrow cells transplantated (BMT) in 9 Gy whole body irradiation (WBI) control mice. (B) Distribution of the GFP-BMDCs in WBI + BMT control mice. (C) Triple stains of CD11b, CD45 and GFP by flow cytometry. (a) The CD45 versus CD11b dot plot of ALTS1C1 tumors. P4 microglia region (CD11b^hi^/CD45^mid^) was indicated as green color and P5 macrophage region (CD11b^hi^/CD45^hi^) was indicated as blue color. (b) The gated P4 region was analyzed for the presence of GFP fluorescent. (c) The gated P5 region was analyzed for the presence of GFP fluorescent. (D) Quantification of the percentage of GFP+ cells in CD11b^hi^/CD45^mid^-microglia and CD11b^hi^/CD45^hi^-macrophage by the histogram analysis as shown in (b) and (c) above. Representative data was from one of ALTS1C1 tumor-bearing brain and three mice were analyzed in each experiment. Symbols and error bars are mean ± SD for n = 3 animals per group.
(DOC)

Figure S3 **The expression of SDF-1 and HIF-1 in invading tumor.** IHC staining of SDF-1 (red), HIF-1 (green), and nuclei by DAPI (blue) on control and 8 Gy single dose irradiated ALTS1C1 brain tumors. Scale bar  = 200 μm.
(DOC)

Method S1 **Bone Marrow Extraction and Transplantation.**
(DOCX)
