# Supplementary material for: Radiation Therapy-Induced Tumor Invasiveness Is Associated with SDF-1-Regulated Macrophage Mobilization and Vasculogenesis
Source: PLoS One. 2013 Aug 5;8(8):e69182. doi: 10.1371/journal.pone.0069182 (PMC3734136; doi:10.1371/journal.pone.0069182)
Supplement: Figure S1 — Histopathology of control (un-disturbed tumor) versus irradiated tumor (A) DAPI stain on frozen section and (B) H&E stain on paraffin section on control or 8 Gy single dose-irradiated ALTS1C1 brain tumors. Scale bar = 100 μm. (DOC) [file pone.0069182.s001.doc]

Figure S1:
