# Supplementary figures and images for: Radiation Therapy-Induced Tumor Invasiveness Is Associated with SDF-1-Regulated Macrophage Mobilization and Vasculogenesis
Source: PLoS One. 2013 Aug 5;8(8):e69182. doi: 10.1371/journal.pone.0069182 (PMC3734136; doi:10.1371/journal.pone.0069182)

Figure S2:


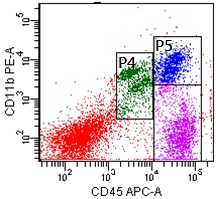


(a)

(b)

(c)

Supplement: Figure S2 — The origin of macrophages in ALTS1C1 brain tumors. (A) Schema of the GFP- bone marrow cells transplantated (BMT) in 9 Gy whole body irradiation (WBI) control mice. (B) Distribution of the GFP-BMDCs in WBI + BMT control mice. (C) Triple stains of CD11b, CD45 and GFP by flow cytometry. (a) The CD45 versus CD11b dot plot of ALTS1C1 tumors. P4 microglia region (CD11bhi/CD45mid) was indicated as green color and P5 macrophage region (CD11bhi/CD45hi) was indicated as blue color. (b) The gated P4 region was analyzed for the presence of GFP fluorescent. (c) The gated P5 region was analyzed for the presence of GFP fluorescent. (D) Quantification of the percentage of GFP+ cells in CD11bhi/CD45mid-microglia and CD11bhi/CD45hi-macrophage by the histogram analysis as shown in (b) and (c) above. Representative data was from one of ALTS1C1 tumor-bearing brain and three mice were analyzed in each experiment. Symbols and error bars are mean ± SD for n = 3 animals per group. (DOC) [file pone.0069182.s002.doc]
