# Supplementary material for: Radiation Therapy-Induced Tumor Invasiveness Is Associated with SDF-1-Regulated Macrophage Mobilization and Vasculogenesis
Source: PLoS One. 2013 Aug 5;8(8):e69182. doi: 10.1371/journal.pone.0069182 (PMC3734136; doi:10.1371/journal.pone.0069182)
Supplement: Figure S3 — The expression of SDF-1 and HIF-1 in invading tumor. IHC staining of SDF-1 (red), HIF-1 (green), and nuclei by DAPI (blue) on control and 8 Gy single dose irradiated ALTS1C1 brain tumors. Scale bar = 200 μm. (DOC) [file pone.0069182.s003.doc]

**Figure S3:**
