# Supplementary material for: Radiation Therapy-Induced Tumor Invasiveness Is Associated with SDF-1-Regulated Macrophage Mobilization and Vasculogenesis
Source: PLoS One. 2013 Aug 5;8(8):e69182. doi: 10.1371/journal.pone.0069182 (PMC3734136; doi:10.1371/journal.pone.0069182)
Supplement: Method S1 — Bone Marrow Extraction and Transplantation. (DOCX) [file pone.0069182.s004.docx]

**Method S1:** *Bone Marrow Extraction and Transplantation*

The experiment mice all obtained from National Laboratory Animal Center, Taiwan. Six- to twelve-week-old C57BL/6-Tg(CAG-EGFP)1Osb/J mice were used as bone marrow donors and six-to eight-week-old C57BL/6J mice as recipients. BM cells from the donors were harvested from the femurs and tibias by flushing the bone cavity with a mixture of RPMI medium and 2% FBS (Invitrogen) using 23-gauge needles (BD, Franklin Lakes, NJ). The recipient mice were lethally irradiated with a dose of 9 Gy 24 hours prior to the BM transplantation as described in previous publication ([Chen, Chiang et al. 2011](#_ENREF_9)). The recipient mice received 5 x 10^6^ GFP-BM cells intravenously and were allowed to recover for 4 weeks.
